# Pre-exercise cryotherapy reduces myoglobin and creatine kinase levels after eccentric muscle stress in young women

**DOI:** 10.3389/fphys.2024.1413949

**Published:** 2024-06-19

**Authors:** Justyna Kusmierczyk, Magdalena Wiecek, Marek Bawelski, Zbigniew Szygula, Katarzyna Rafa-Zablocka, Malgorzata Kantorowicz, Jadwiga Szymura

**Affiliations:** ^1^ Department of Physiology and Biochemistry, University of Physical Education in Kraków, Kraków, Poland; ^2^ Department of Sports Medicine and Human Nutrition, University of Physical Education in Kraków, Kraków, Poland; ^3^ Department Brain Biochemistry, Maj Institute of Pharmacology, Polish Academy of Sciences, Kraków, Poland; ^4^ Medical Institute, University of Applied Sciences, Nowy Targ, Poland; ^5^ Department of Clinical Rehabilitation, University of Physical Education in Kraków, Kraków, Poland

**Keywords:** whole-body cryotherapy, eccentric works, muscle damage, myoglobin, creatine kinase

## Abstract

**Introduction:** The aim of this study was to investigate the effect of pre-exercise whole-body cryotherapy (WBC) on muscle damage indicators following eccentric treadmill exercise in young women.

**Methods:** Twenty-seven participants underwent two 1-h downhill treadmill runs, replicating 60% of their maximal oxygen uptake, with a 4-week intermission for recovery and treatment application. In this intermission, one group underwent 20 sessions of WBC, delivered five times a week at −120°C for 3 min each, while the comparison group received no such treatment. Markers of muscle injury—serum myoglobin concentration, creatine kinase and lactate dehydrogenase activity and also uric acid, and cell-free DNA concentration—were measured before and after downhill runs.

**Results:** The study observed a notable reduction in post-exercise myoglobin and CK levels in the WBC group after the second running session.

**Discussion:** The results suggest that WBC can have a protective effects against muscle damage resulting from eccentric exercise.

## 1 Introduction

Whole-body cryostimulation (WBC), medically denominated as whole-body cryotherapy, encompasses succinct exposures (not exceeding 3 min) to frigid ambient conditions, with temperature parameters setting between −100°C and −160°C. As the research review shows, these safe, rarely causing side effects cold-temperature treatments (Legrand et al., 2023), are deployed in order preventative health, therapeutic regimens as well as regeneration and sport medicine (Lombardi et al., 2017; Patel et al., 2019; Bouzigon et al., 2021; Garcia et al., 2021; Kujawski et al., 2021; Fontana et al., 2022; Dziedzic et al., 2024; Jeyaraman et al., 2024).

The scholarly dissemination on WBC’s implementation within the field of women’s sports medicine remains inchoate as compared to the entirety of its viable applications. The investigative emphasis is traditionally directed at its utilization for recuperation post physical training or throughout an athletic season, while inquiry into its “prophylactic” or stimulating influences via repeated WBC sessions upon biochemical markers is notably scant (Szymura et al., 2018; Wojciak et al., 2020; Zembron-Lacny et al., 2020).

From previous research we know that extremely low temperatures incite a prompt stress reaction within cellular structures (Vieira Ramos et al., 2016). Upon prolonged exposure to such stressors, it is posited that cellular responses evolve in conjunction with adaptive processes (Barqawi et al., 2021). Therefore, when studying the effect of cryotemperatures on the body, the duration of exposure must be considered (Lubkowska et al., 2012). In animal muscle cells, after cryotherapy, inflammatory processes after injury were found to be attenuated by regulating the expression of mRNA for tumor necrosis factor alpha (TNF-a), nuclear factor-κB (NF-κB), transforming growth factor beta (TGF-β) and matrix metalloproteinase 9 (MMP-9) and a reduction in the percentage of macrophages (Vieira Ramos et al., 2016). In human, WBC significantly reduced the post-exercise generation of reactive oxygen and nitrogen species (H_2_O_2_ and NO), and the concentrations of serum interleukin 1β and C-reactive protein (Zembron-Lacny et al., 2020), as well as increased sirtuins concentration (Sirt1, Sirt3) and antioxidative capacity (Wojciak et al., 2020).

It was found that WBC induce changes in both innate and adaptive branches of the immune system, hormones, and metabolic status in non-professional male athletes, suggesting a beneficial involvement of WBC in tissue repair (Nasi et al., 2020), but hepatocyte growth factor, insulin-like growth factor, platelet-derived growth factor, vascular endothelial growth factor, and brain-derived neurotrophic factor, were also reduced by WBC exposure (Zembron-Lacny et al., 2020).

The primary response to whole-body cryotherapy (WBC) involves vasoconstriction in the skin and subcutaneous tissue due to the stimulation of α2-adrenergic receptors (Charkoudian, 2003). This results in a redistribution of blood flow and a decrease in skin temperature (Skrzek et al., 2019), followed by reperfusion and a sudden normalization of skin temperature (Westerlund et al., 2003). Subsequent effects include anti-inflammatory and antioxidant responses.

There are gender differences in the response to WBC. The change in individuals” skin temperature in response to WBC depends on anthropometric variables, with individuals having greater obesity and a larger body surface area to body mass ratio cooling more than lean individuals (Hammond et al., 2014). After WBC, women experience a greater decrease in average skin temperature compared to men, showing a negative correlation with body fat content (Cuttell et al., 2017). Sex differences in cellular stress responses across endocrine, inflammatory, and redox pathways were observed (Hodes et al., 2024). During adaptations to cold stress, men predominantly used strategies involving greater metabolic and shivering responses, while women demonstrated more effective insulating responses (Solianik et al., 2014). It was implied that cold stress causes an increase in the level of Unc-51-like kinase-1c, associated with the autophagy initiation process, only in peripheral blood mononuclear cells isolated from men, suggesting different cytoprotective mechanisms between the sexes (King et al., 2024). Despite the similar impacts on resting energy expenditure during cold exposure, more significant changes in plasma glucose, leptin, and adiponectin levels were observed in women (Mengel et al., 2020). Cooling-induced stress was found to affect both genders similarly in terms of central and peripheral fatigability, yet it predominantly reduced fatigue in males during sustained maximal voluntary contractions (Solianik et al., 2015; Castellani and Young, 2016). Enhanced glucocorticoid secretion in response to stress was exhibited by females, attributed to a differently regulated hypothalamic-pituitary-adrenal axis (Handa and Weiser, 2014; Heck and Handa, 2019). Moreover, it was noted that female mitochondria produce less hydrogen peroxide and contain higher levels of antioxidant enzymes compared to those in males (Mendoza-Núñez et al., 2010; Miotto et al., 2018; Silaidos et al., 2018).

Eccentric exercise, which involves lengthening of the muscle under tension, can lead to muscle damage and oxidative stress (Wiecek et al., 2017).

WBC triggers adaptive processes within the body, leading to improved cellular responses and overall recovery (Vieira Ramos et al., 2016). In addition, WBC has been found to reduce muscle pain and inflammation, improve athletic performance and capacity, and enhance recovery from muscle damage (Capodaglio et al., 2022). It is important to note that the existing body of research primarily focuses on male subjects. However, as previously shown, eccentric exercise causes oxidative damage to lipids in women, which indicates a redox balance disturbance (Wiecek et al., 2017). In men, eccentric submaximal aerobic exercise does not induce oxidative stress. These results, as well as gender differences in response to cryogenic temperatures (Cuttell et al., 2017), highlights a need for more inclusive studies involving female participants to fully understand the potential benefits of WBC in preventing muscle damage and promoting recovery in individuals of all genders.

The aim of our study is to determine whether the pre-exercise use of 20 WBC treatments can mitigate muscle damage and pain after aerobic eccentric exercise in untrained women, as well as whether it can expedite muscle recovery. In our studies, we indirectly assess myocyte damage based on changes in biochemical markers in the blood (myoglobin, creatine kinase, lactate dehydrogenase, uric acid, cfDNA) and subjective assessment of pain perception.

We hypothesize that the level of myocyte damage and pain is reduced and the rate of recovery from aerobic eccentric exercise is greater following 20 WBC treatments.

## 2 Materials and methods

### 2.1 Study design

The investigative study comprised of 27 female participants between the age of 19–25 years. Selection was based on exhaustive medical assessments, involving blood morphology, lipid profiling, glucose concentrations, glycated hemoglobin (HbA1c) levels, arterial blood pressure readings, and electrocardiogram evaluations. Exclusion criteria included previous exposure to WBC procedures within the preceding 6 months, medical contraindications for WBC, or the necessity for uninterrupted pharmacological treatment.

*A priori* power analysis was performed utilizing G*Power 3.1 software (Dusseldorf, Germany) to determine the requisite sample size for the study (Faul et al., 2007). Considering the experiment’s structure of two groups and eight measurement points, alongside a predefined alpha of 0.05 and a test power of 0.95, it was calculated that a total of 24 subjects (twelve per group) would be required to adequately power the study.

Randomization ensued, forming two distinct cohorts: a control group and one subjected to WBC interventions. An exercise test was administered to deduce the maximal oxygen consumption (VO_2_max) of each participant and to establish exercise intensity for a 60-min downhill treadmill run at 60% VO_2_max. This specific exercise modality was selected to provoke eccentric muscular effort, which instigates distinct muscular responses compared to concentric activity. Biomarker samples to gauge muscle damage were systematically drawn before the exercise, 5 min, 60 min, and 24 h subsequent to the exertion.

Post-evaluation, one subset of 14 individuals underwent a series of twenty WBC sessions, involving 3-min exposures at −120°C, while the rest functioned as a control group. The influences of cryotherapy on the biochemical markers related to muscle and oxidative stress, initiated by the eccentric exercise, were analyzed. A follow-up downhill run was conducted subsequent to the last WBC session. Participants were instructed to maintain their habitual dietary and physical activity routines throughout the duration of the study.

### 2.2 Study participants

Prospective subjects with health concerns precluding WBC utilization, previous WBC users within 6 months, smokers, individuals exhibiting substance abuse, those currently on medication, athletes, and women with irregular menstruation cycles were excluded.

**FIGURE 1:**
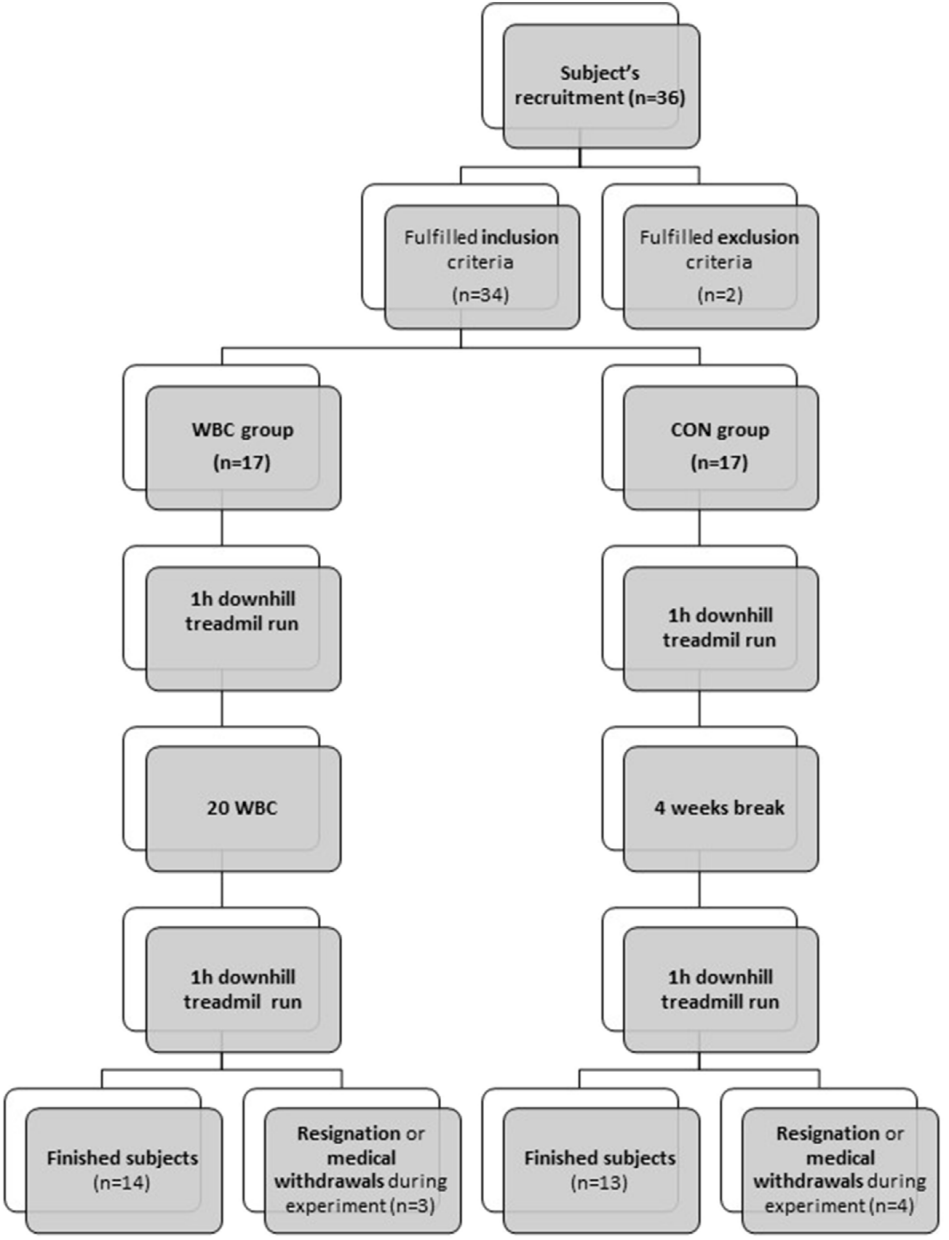
Flow chart of participants.

The study began with 36 eligible candidates, 34 of whom were included; however, attrition led to seven withdrawals during the investigation. The final composition of the study population included 27 women, partitioned as follows: The WBC group, which participated in cryotherapy sessions between downhill running activities (*n* = 14). The control group, which abstained from WBC engagements (*n* = 13). The flow chart of participants is presented at Figure 1.

The research protocol adhered rigorously to the ethical guidelines set forth in the Declaration of Helsinki and received approval from the Bioethical Committee of the Regional Medical Chamber (55/KBL/OIL/2022).

### 2.3 Procedure of whole-body cryotherapy

The WBC protocol was executed as follows: Participants initially experienced a 30-s episode within a −60°C antechamber, subsequent to which they were exposed for 3 min to −120°C temperatures in the main cryogenic chamber. The cooling of the chamber air was facilitated through the employment of liquid nitrogen. The KN-1 model cryogenic chamber, hailing from the Bamet company of Wielka Wies, Poland, served as the locale for these sessions. Oxygen levels within the chamber were rigorously kept within the 21%–22% range, a statistic confirmed by continuous monitoring facilitated by dual oxygen sensors, specifically the EurOx.O2 G/E models from Krakow, Poland.

The treatment protocol accommodated up to four participants per session, who were instructed to ambulate in a circular pattern within the chamber. Upon audio cues, the direction of motion was reversed, ensuring participants followed a consistent and controlled respiratory pattern throughout the exposure.

In terms of attire, participants donned athletic shorts, sports bra, knee-high socks, clogs, gloves, ear-caps, and surgical masks with gauze covering the oral and nasal regions. All accessories, inclusive of eyeglasses and watches, were mandatorily removed preceding chamber entry. Moisture elimination and non-abrasive skin contact were emphasized to mitigate the risk of frostbite during treatment.

The cryotherapy installation featured an intricate array of equipment encompassing real-time temperature monitoring systems for both chambers, an automatic air drying function, oxygen concentration sensors, and an interior chamber surveillance via video feed for continual observation. Additionally, the facility was equipped with an emergency button and instantly operable door release mechanism for user safety. Observation through thermally insulated windows and communication through a camera system enabled direct supervision. Certified physical therapists oversaw each treatment session, ensuring adherence to safety protocols and procedural integrity.

### 2.4 Assessment of body composition

Prior to initiating the sequence of running sessions, body height and body mass of each participant were documented. Subsequent to these measurements, body composition was assessed via electrical bioimpedance analysis using the Jawon IOI-353 Body Composition Analyzer (Gyeongsa, Korea)—a multifrequency bioelectrical impedance device—equipped with eight electrodes functioning at frequencies of 5, 50, and 250 kHz. The body composition data obtained through this method is provided in Table 1.

**TABLE 1:** Body composition and blood pressure.

Variable	CON	WBC	*p*-value
BM (kg)	63.00 ± 7.14	65.12 ± 8.34	0.61
BH (cm)	166.21 ± 5.93	165.39 ± 5.02	0.56
BF (%)	26.45 ± 5.15	27.63 ± 4.60	0.81
LBM (kg)	44.76 ± 3.81	43.57 ± 3.51	0.73
SBP (mmHg)	120 ± 5.02	120 ± 7.82	0,98
DBP (mmHg)	75 ± 6.04	75 ± 4.67	0,96

Values are means ± SD; BM, body mass; BH, body height; BF, body fat; LBM, lean body mass; SBP, systolic blood pressure; DBP, diastolic blood pressure; CON, control group; WBC, Whole-body cryotherapy group.

### 2.5 The graded test protocol

The protocol for graded exercise employed in this study aimed to ascertain each subject’s maximal oxygen consumption (VO_2_max). This was pivotal in setting the specific workload for the ensuing 60-min treadmill (h/p/Cosmos Saturn, Germany) descent at 60% of the individual’s VO_2_max, as delineated in the previous work by Wiecek et al. (2017). Commencing with a 0° treadmill incline, the exercise began at a pace of 6 km/h, with a subsequent speed increment of 1.0 km/h every 2 min. This increment was upheld until the participant either elected to terminate the test (volitional exhaustion) or did not manifest any additional rise in oxygen uptake despite escalating exercise intensity.

Throughout the graded test, vital cardiorespiratory metrics were monitored, including, but not limited to, oxygen consumption, production of carbon dioxide, the respiratory exchange ratio, and heart rate. These were performed via a MetaLyzer 3B from Cortex, Germany, and a Polar H10 heart rate sensor from Polar Electro, Finland. The criteria for determining VO_2_max were stringent as previously described (Wiecek et al., 2017), calling for an observable plateau in oxygen consumption concomitant with an RER in excess of 1.1, alongside the achievement of a heart rate nearing the subject’s age-predicted ceiling by a margin of 10 beats per minute.

### 2.6 Execution of the downhill running task

The downhill running exercise was conducted at a −10% incline on a treadmill, initially set at a speed of 6 km/h. Following a 4-min period, the participants” pace was adjusted to meet 60% of their VO_2_max, gauged with precision. This velocity was sustained for the duration of 1 h, during which cardiorespiratory parameters were consistently measured.

### 2.7 Blood sample collection protocol

**FIGURE 2:**
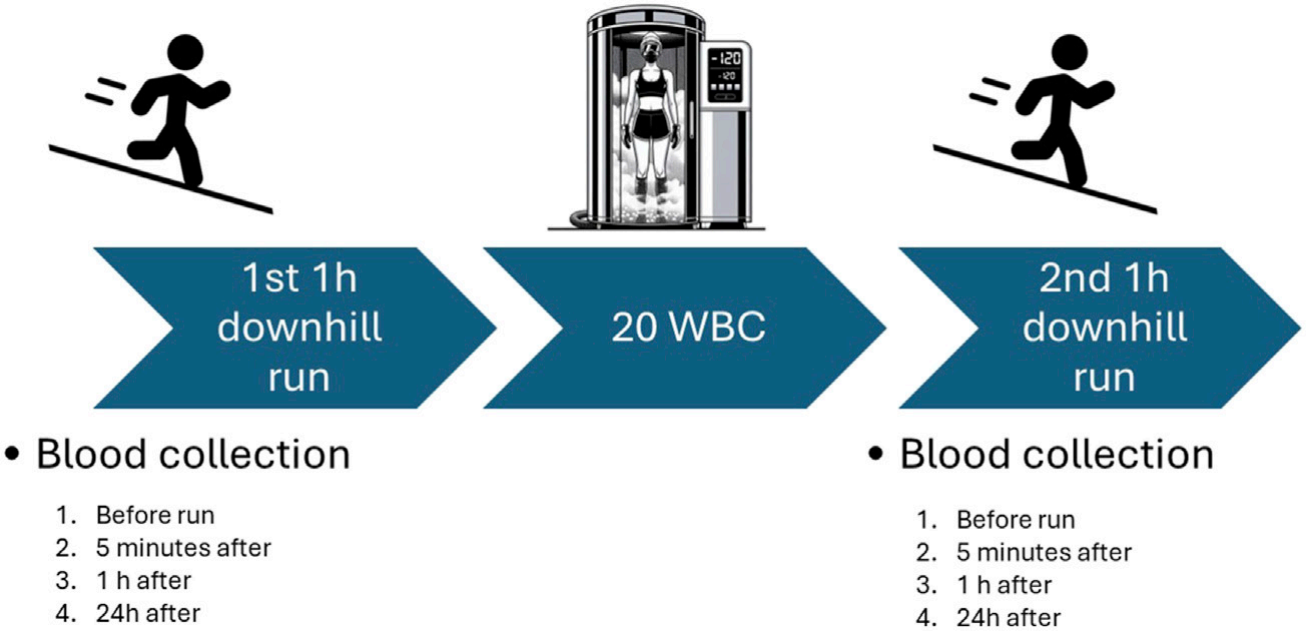
Study scheme.

Venipuncture was conducted at eight distinct time points throughout the study: before the initial downhill exercise (1 pre), 5 min after (1 post), at 1 hour post-exercise (1 post 1 h), and at 24 h post the first exercise bout (1 post 24); and similarly timed surrounding the second bout of exercise (2 pre, 2 post, 2 post 1 h, and 2 post 24). For these procedures, a vacuum extraction methodology was employed, utilizing equipment provided by Becton Dickinson, established in Franklin Lakes, NJ, United States. Participants were seated and allowed to rest for 5 min prior to the blood draw. The participant could not eat drink after the first blood collection till 1 h after the run. A schematic diagram illustrating the blood collection protocol is presented in Figure 2. For the serum and plasma collections, blood was collected into specialized 4 mL Vacutainer™ tubes fitted with clot activators for serum retrieval, and K2EDTA for plasma extraction. Subsequent to collection, these samples were centrifuged for a quarter hour at an RCF of 1,000 × g. The centrifuge equipment utilized was the MPW-351R (MPW Med. Instruments of Warsaw, Poland), which operates at a controlled temperature of 4 C. Post-centrifugation, the serum and plasma specimens were preserved at −80°C ± 5 C using the ZLN-UT 300 PREM freezer from POL-EKO-APARATURA, Wodzisław Śląski, Poland, thus retaining their integrity until it was time for analysis.

To measure lactate concentration, capillary blood was taken from participants both before and immediately after succeeding the exercise bouts. Specimen collection was carried out using Microvette^®^ fluoride/heparin tubes, which incorporate a glycolysis inhibitor to preserve sample integrity. The centrifugation of these samples was performed at ambient temperature (21 C) for 3 min at an RCF of 14,300 × g with the MPW-55 centrifuge.

### 2.8 Pain self assessment

Following each blood collection, participants were instructed to perform a squat, which served as a stimulus for the pain assessment process. They then evaluated and communicated the level of pain they experienced using the Visual Analogue Scale. The VAS is a validated tool designed to measure pain intensity—a subjective experience. It employs a 10-cm line as a continuum where the endpoints are defined as “no pain”—white color and “worst pain imaginable”—dark red color. Individuals report their pain by marking a colored point on the line that corresponds to the intensity of their pain, offering a quantifiable measure of pain levels for both acute and chronic conditions.

### 2.9 Biochemical analysis

The biochemical analysis for this study involved the measurement of lactate and uric acid concentrations, as well as the activity of creatine kinase and lactate dehydrogenase. These parameters were evaluated using spectrophotometric methods, with assay kits provided by RANDOX (catalog numbers: LC2389 (plasma level 0.5–1.6 mmol/L; sensitivity 0.155 mmol/L, Intra assay CV(%): 2.07, Inter Assay CV(%): 1.24), UA230 (serum level in women 142–339 μmol/L; sensitivity 36.2 μmol/L, Intra assay CV(%): 1.77, Inter Assay CV(%): 3.95), CK110 (serum level 24–175 U/L, sensitivity 21.7 U/L, Intra assay CV(%): 2.31, Inter Assay CV(%): 3.91), and LD401 (serum level 230–460 U/L, sensitivity 55 U/L, Intra assay CV(%): 3.86, Inter Assay CV(%): 3.99); RANDOX Global Healthcare, United Kingdom). The detection of these measurements was carried out on an Evolution™ 201 UV-Visible Spectrophotometer (Thermo Scientific™, located in MA, United States).

Myoglobin concentration was determined separately using an enzyme-linked immunosorbent assay with the DRG ELISA kit (serum level 12–100 ng/mL; sensitivity 5 ng/mL, Intra assay CV(%): 3.9, Inter Assay CV(%): 7.8) (DRG International, NJ, United States). The detection in this case was performed using the SPARK™ Microplate Reader (Tecan Group Ltd., Switzerland).

Additionally, cell-free DNA (cfDNA) concentrations were assessed. The process began by isolating cfDNA from a 300 μL plasma sample using the Higher Purity circulating DNA Purification kit (Canvax, Spain). Subsequent quantification of cfDNA was then realized through the application of the Pico488 ds DNA Quantification kit (Lumiprobe GmbH, Germany), in accordance with the guidelines prescribed by the manufacturer.

The results of CK, LDH activity and cfDNA, lactate, myoglobin and UA concentration, were corrected by the percentage changes in plasma volume (%1PV) according to the Kraemer and Brown formula (Kraemer and Brown, 1986). ΔPV was calculated on the basis of changes in HGB concentration (g/dL) and HCT values (%) (Dill and Costill, 1974; Harrison et al., 1982).

### 2.10 Statistical analysis

All statistical analyses were conducted using the STATISTICA 13.3 software package (TIBCO, Inc., CA, United States). The normality of the distribution of the variables was tested with the Shapiro–Wilk test, and the homogeneity of variances was confirmed with Levene’s test. Depending on whether the data was normally distributed or not, different statistical tests were employed. For variables with a normal distribution, one-way ANOVA and Student’s t-test were used, while for those not normally distributed, the non-parametric Kruskal-Wallis and Friedman test were utilized.

To evaluate the impact of whole-body cryotherapy and the timing of venipuncture on various outcome variables, a repeated measures ANOVA was used. This test helps in understanding the main effects of factors such as the Group (whether they were in the Control or WBC group) and Time, as well as any interaction effect between Group and Time. If significant effects were found, *post-hoc* analyses were conducted using the Tukey test to further investigate the differences.

For all the tests, a *p*-value of 0.05 or below was set as the criterion for statistical significance. This means that if the observed differences had a *p*-value of less than or equal to 0.05, they were considered statistically significant.

## 3 Results

### 3.1 Body composition

The somatic build are presented in Table 1. There were no differences between the groups.

### 3.2 Blood morphology

Blood morphology results are in Table 2.

**TABLE 2:** Blood morphology.

Variable	CON	WBC	*p*-value
RBC (10^6^/µL)	4.48 ± 0.21	4.56 ± 0.18	0.81
HGB (g/dL)	13.43 ± 0.46	13.54 ± 0.72	0.93
HCT (%)	38.2 ± 2.24	39.24 ± 1.62	0.91
PLT (10^3^/µL)	255.2 ± 41.4	253.9 ± 48.6	0.92
LEUC (10^3^/µL)	5.63 ± 1.23	5.49 ± 1.21	0.93
NEUT (%)	47.83 ± 6.24	48.34 ± 6.41	0.87
LYMPH (%)	35.16 ± 5.34	36.42 ± 5.43	0.89
MONO (%)	9.43 ± 2.42	9.37 ± 2.34	0.92
EOS (%)	2.63 ± 1.14	2.46 ± 1.39	0.88
BASO (%)	0.56 ± 0.42	0.63 ± 0.54	0.93

Values are means ± SD; RBC, red blood cells; HGB, hemoglobin; HCT, hematocrit; PLT, platelets; LEUC, leukocytes; NEUT, neutrophils; LYMPH, lymphocytes; MONO, monocytes; EOS, eosinophils; BASO, basophils; CON, control group; WBC, Whole-body cryotherapy group.

### 3.3 Physiological parameters and lactate level

In Table 3 there are results of graded test, calculation for downhill run and lactate concentration from the downhill runs. There were no significant differences between the CON and WBC group.

**TABLE 3:** Physiological parameters and lactate level.

Variables	CON	WBC	*p*-value
VO2max [l/min]	2.49 ± 0.29	2.63 ± 0.50	0.38
VO2max [ml/(kg × min)]	42.33 ± 4.74	42.14 ± 5.87	0.92
60% VO2max [l]	1.49 ± 0.17	1.57 ± 0.30	0.39
60% VO2max [ml]	25.40 ± 2.84	25.29 ± 3.52	0.92
Vdownill run [km/h]	9.27 ± 1.64	9.49 ± 1.39	0.70
VEmax [l/min]	87.62 ± 14.63	95.74 ± 20.36	0.28
HRmax [1/min]	193.62 ± 5.19	194.21 ± 6.39	0.79
LA 1 Pre [mmol/l]	2.47 ± 1.07	2.49 ± 1.18	0.96
LA 1 3ʹ [mmol/l]	2.89 ± 2.08	3.37 ± 2.57	0.73
LA 2 Pre [mmol/l]	1.64 ± 0.58	1.64 ± 1.09	0.98
LA 2 3ʹ [mmol/l]	1.44 ± 0.98	1.70 ± 1.01	0.88

Values are means ± SD VO2max, maximal oxygen uptake; Vdownill run, velocity of downhill run; VEmax, maximal pulmonary ventilation; HRmax, maximal heart rate; LA, lactate concentration before both downhills run and 3 min after the runs; CON, control group; WBC, Whole-body cryotherapy group.

### 3.4 Biochemical markers

#### 3.4.1 Myoglobin concentration

**FIGURE 3:**
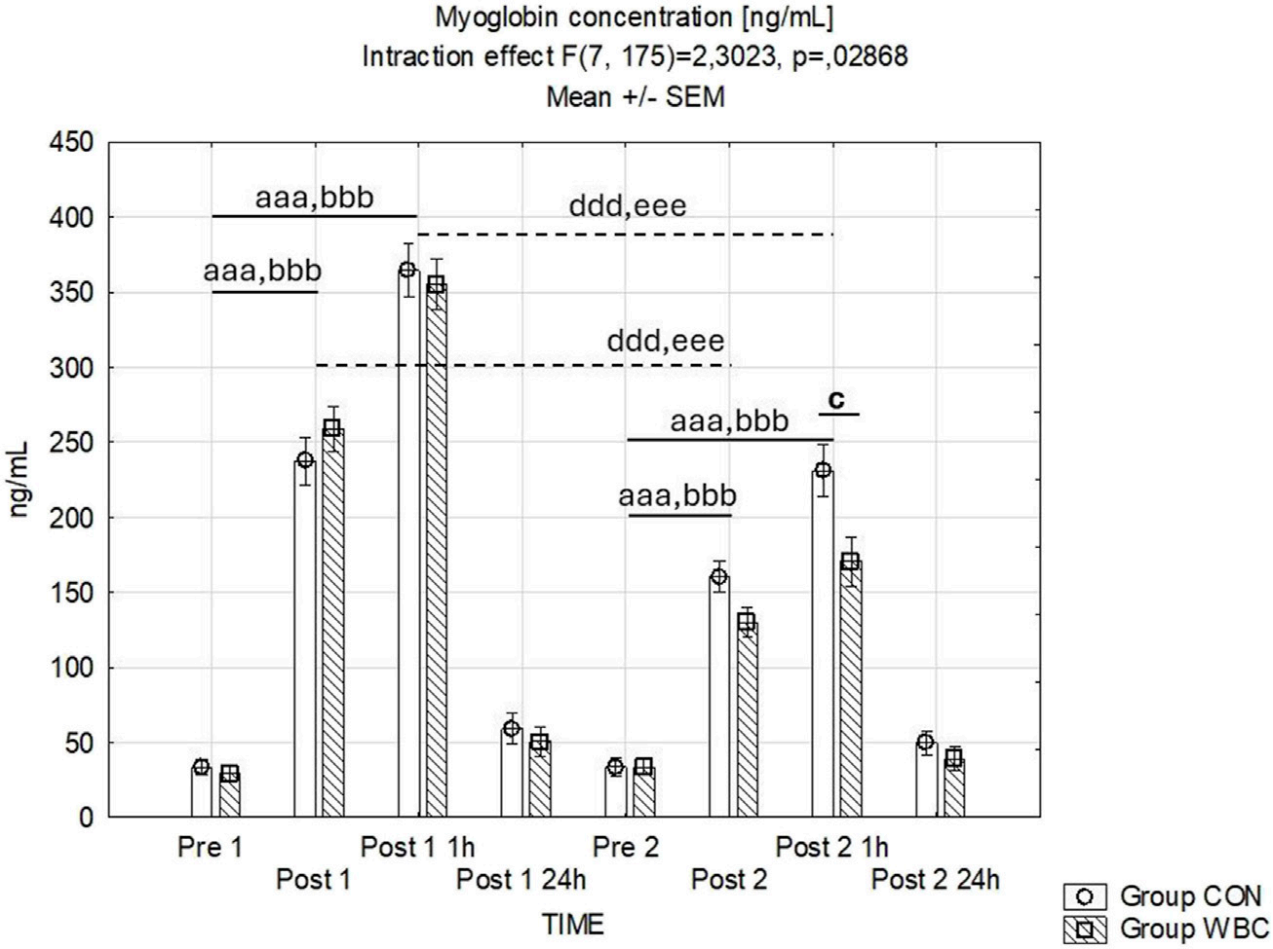
Myoglobin concentration. Myoglobin concentrations (mean ± SEM) in serum for the control (CON) and whole-body cryotherapy (WBC) groups was measured at the following time points: before the first run (Pre 1), 5 min after the first run (Post 1), 1 h after the first run (Post 1 1 h), 24 h after the first run (Post 1 24 h), before the second run (Pre 2), 5 min after the second run (Post 2), 1 h after the second run (Post 2 1 h), and 24 h after the second run (Post 2 24 h). aaa *p* ≤ 0.001 from corresponding pre-exercise values in CON group; bbb *p* ≤ 0.001 from corresponding pre-exercise values in WBC group; c *p* ≤ 0.05 from corresponding CON group values; ddd *p* ≤ 0.001 from corresponding Post 1 CON group values; eee *p* ≤ 0.001 from corresponding Post 1 values WBC.

An interaction was observed between the temporal measurement points and the treatment groups with respect to serum myoglobin levels. An immediate and persistent elevation in myoglobin levels was discernible in both groups immediately and 1 h subsequent to the both downhill running tasks (*p* < 0.001). Notably, the rise in myoglobin concentration observed post-exercise was less pronounced following the second run compared to the first (*p* < 0.001 upon comparing data points immediately after and 1 h subsequent to each run). Furthermore, a diminution in myoglobin concentration was recorded in the WBC group 1 h post-second run compared to the control group (*p* = 0.03) as depicted in Figure 3.

#### 3.4.2 CK activity

**FIGURE 4:**
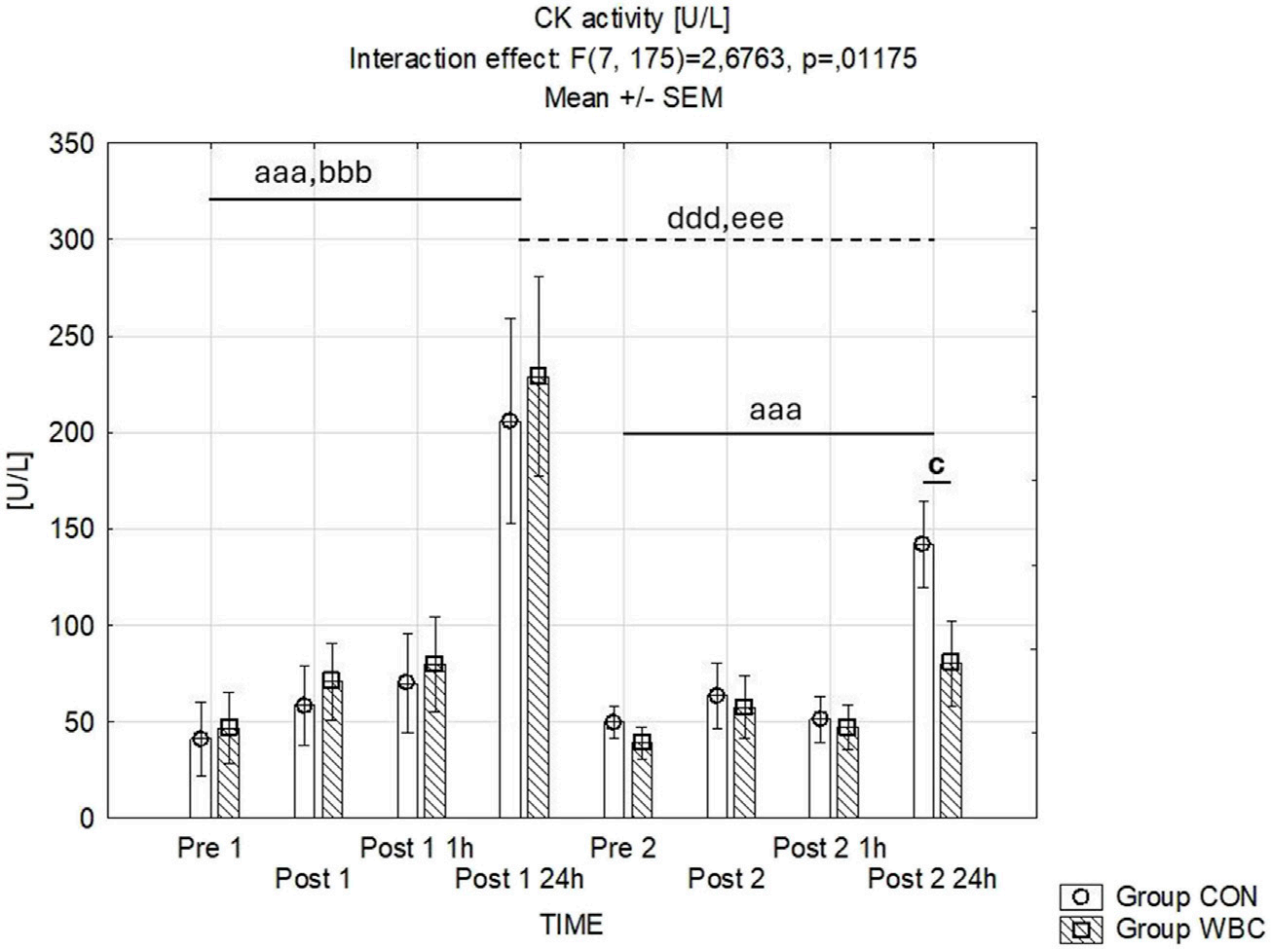
CK activity. Creatinine kinase (CK) activity (mean ± SEM) in serum for the control (CON) and whole-body cryotherapy (WBC) groups was measured at the following time points: before the first run (Pre 1), 5 min after the first run (Post 1), 1 h after the first run (Post 1 1 h), 24 h after the first run (Post 1 24 h), before the second run (Pre 2), 5 min after the second run (Post 2), 1 h after the second run (Post 2 1 h), and 24 h after the second run (Post 2 24 h). aaa *p* ≤ 0.001 from corresponding pre-exercise values in CON group; bbb *p* ≤ 0.001 from corresponding pre-exercise values in WBC group; c *p* ≤ 0.05 from corresponding CON group values; ddd *p* ≤ 0.001 from corresponding Post 1 CON group values; eee *p* ≤ 0.001 from corresponding Post 1 values WBC.

Differential CK activities are delineated in Figure 4. Time-dependent interactions with group treatments were identified. The first downhill runs prompted substantial elevations in CK activity 24 h post-exercise in both cohorts (*p* < 0.001 after the initial run for both groups). Twenty fourhours after the second run, there were only significant increase in CK activity in CON group (*p* < 0.001). Additionally, a lower CK activity was observed 24 h post-second run in the WBC group compared to CON (*p* = 0.038).

#### 3.4.3 LDH activity

**FIGURE 5:**
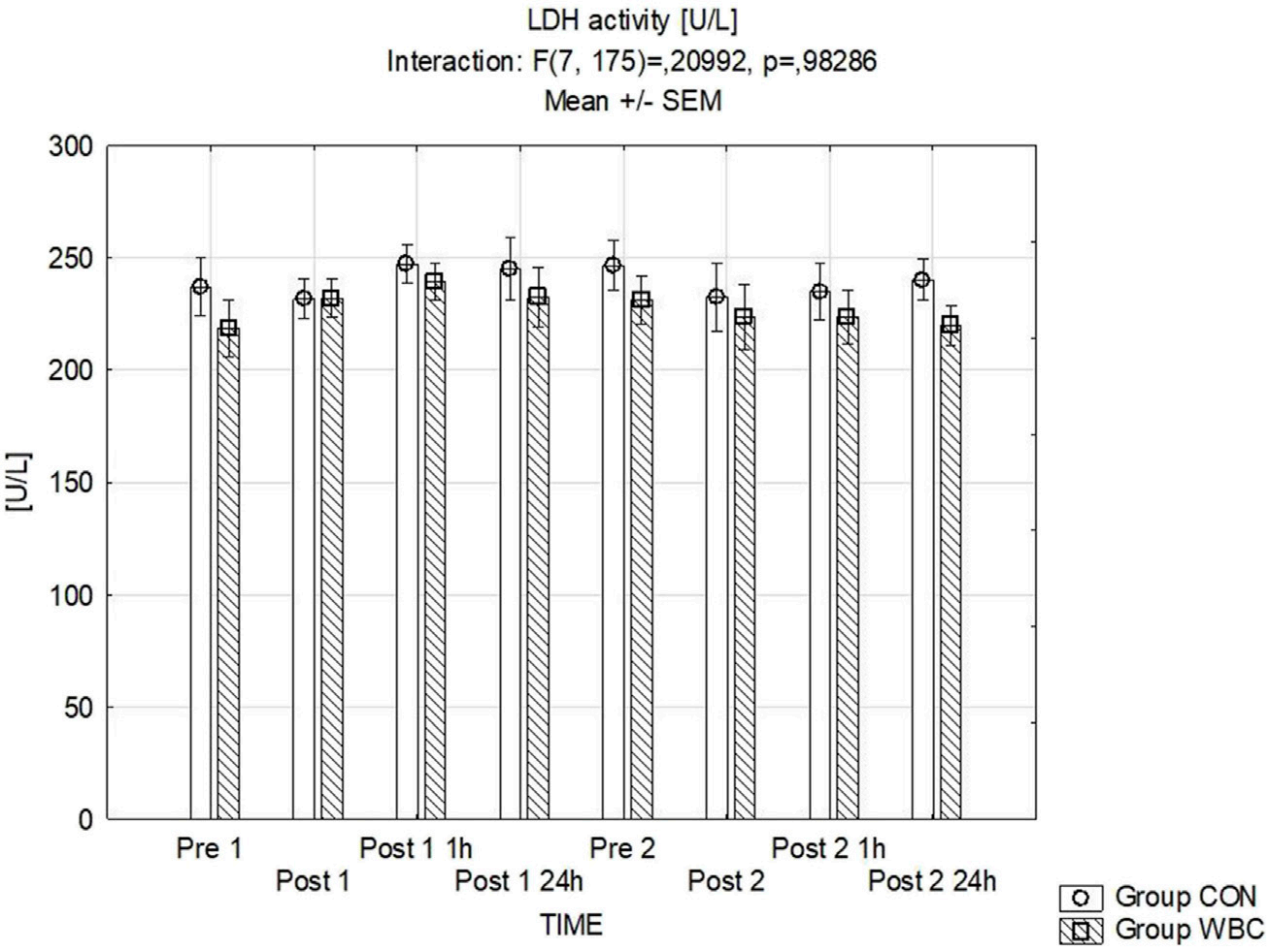
LDH activity. Lactate dehydrogenase (LDH) activity (mean ± SEM) in serum for the control (CON) and whole-body cryotherapy (WBC) groups was measured at the following time points: before the first run (Pre 1), 5 min after the first run (Post 1), 1 h after the first run (Post 1 1 h), 24 h after the first run (Post 1 24 h), before the second run (Pre 2), 5 min after the second run (Post 2), 1 h after the second run (Post 2 1 h), and 24 h after the second run (Post 2 24 h).

The serum LDH activity levels are detailed in Figure 5. Statistical analysis revealed no significant discrepancies.

#### 3.4.4 UA concentration

**FIGURE 6:**
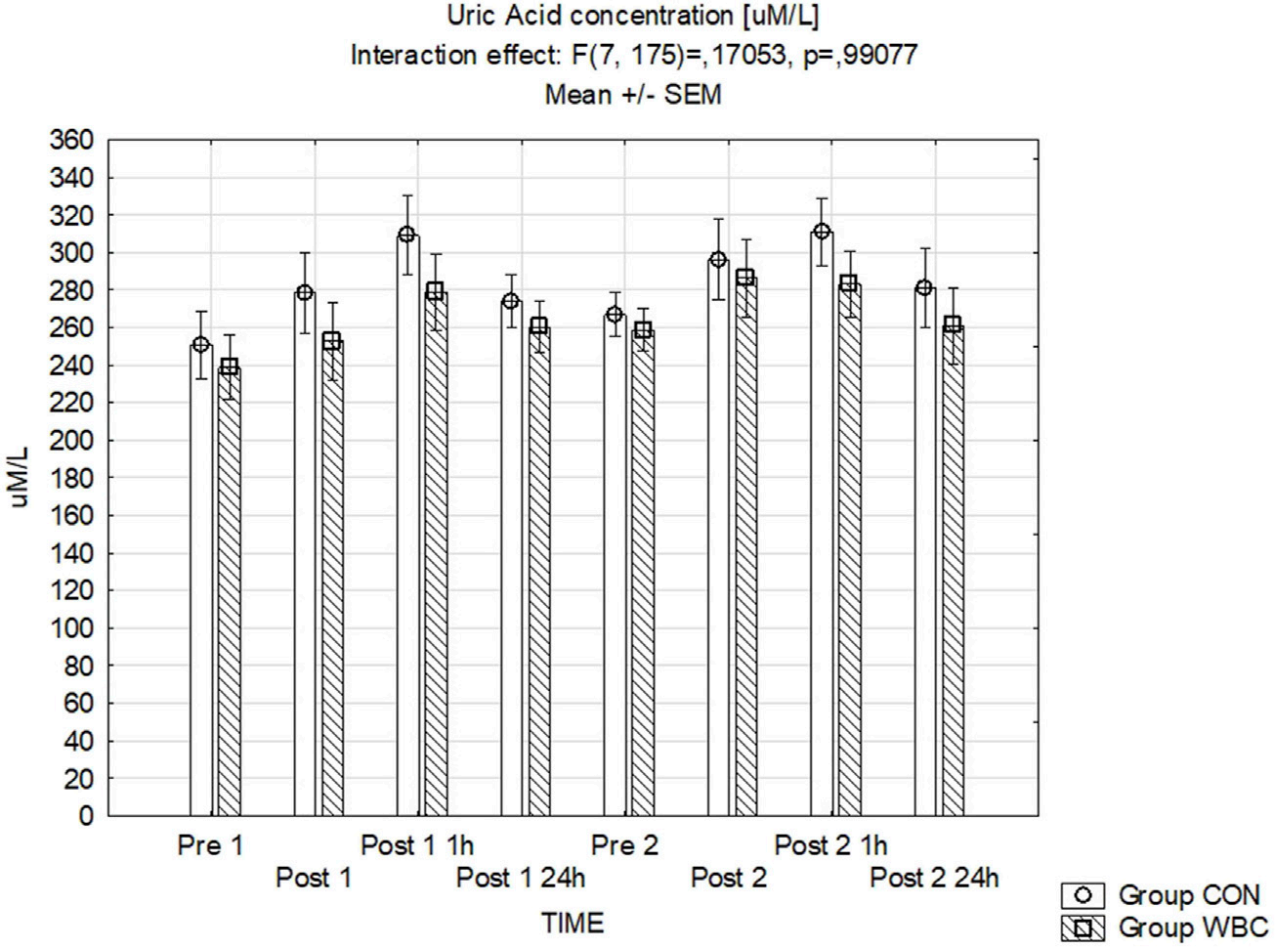
UA concentration. Uric acid (UA) concentration (mean ± SEM) in serum for the control (CON) and whole-body cryotherapy (WBC) groups was measured at the following time points: before the first run (Pre 1), 5 min after the first run (Post 1), 1 h after the first run (Post 1 1 h), 24 h after the first run (Post 1 24 h), before the second run (Pre 2), 5 min after the second run (Post 2), 1 h after the second run (Post 2 1 h), and 24 h after the second run (Post 2 24 h).

Figure 6 presents the data for uric acid concentrations where no significant variations between the groups were detected.

#### 3.4.5 cfDNA concentration

**FIGURE 7:**
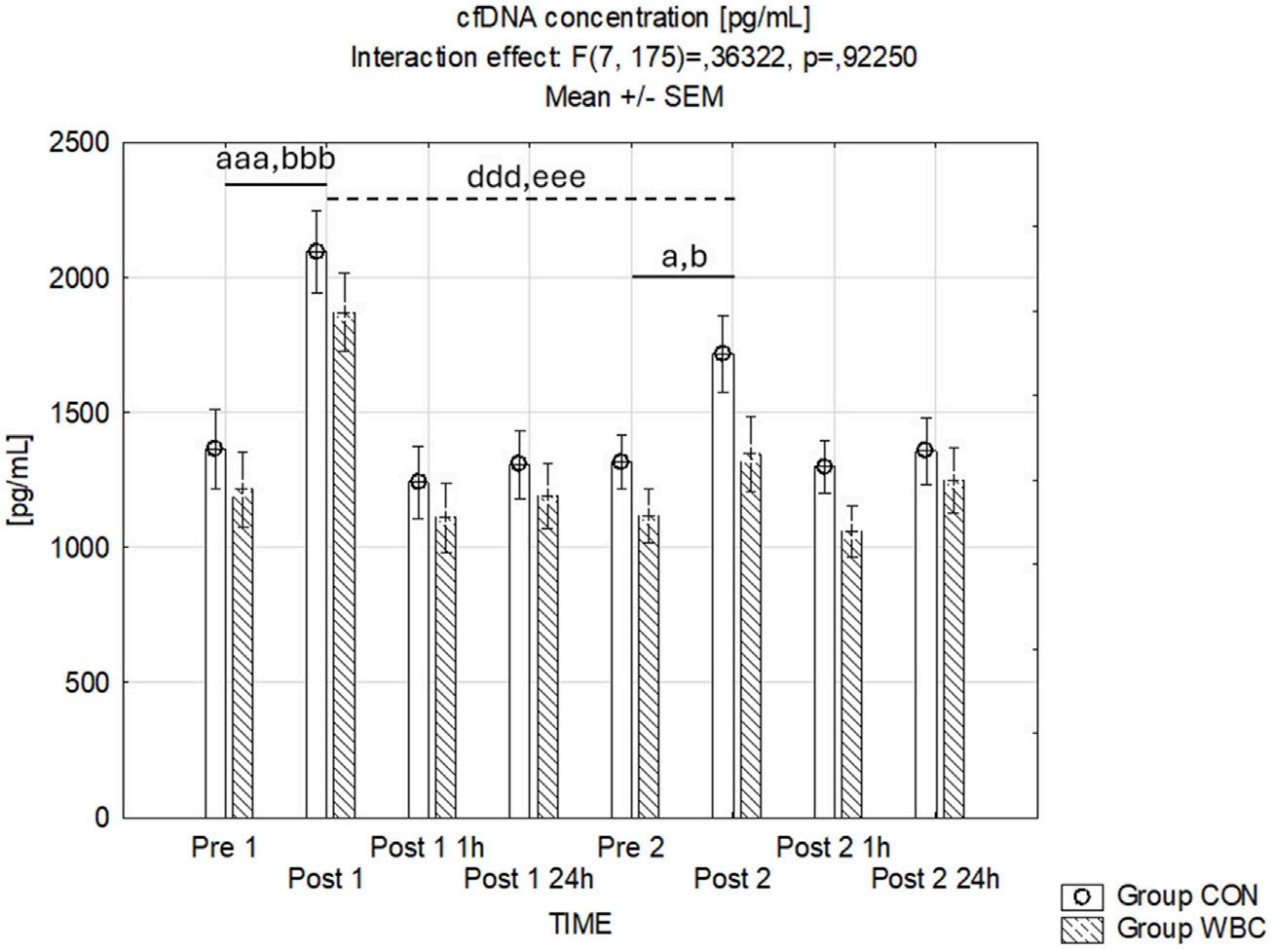
cfDNA Concentration. cfDNA concentrations (mean ± SEM) in serum for the control (CON) and whole-body cryotherapy (WBC) groups was measured at the following time points: before the first run (Pre 1), 5 min after the first run (Post 1), 1 h after the first run (Post 1 1 h), 24 h after the first run (Post 1 24 h), before the second run (Pre 2), 5 min after the second run (Post 2), 1 h after the second run (Post 2 1 h), and 24 h after the second run (Post 2 24 h). a *p* ≤ 0.05 from corresponding pre-exercise values in CON group; b *p* ≤ 0.05 from corresponding pre-exercise values in WBC group; aaa *p* ≤ 0.001 from corresponding pre-exercise values in CON group; bbb *p* ≤ 0.001 from corresponding pre-exercise values in WBC group; ddd *p* ≤ 0.001 from corresponding Post 1 CON group values; eee *p* ≤ 0.001 from corresponding Post 1 values WBC.

Variations in plasma cfDNA concentrations are summarized in Figure 7. The influence of the time-group interaction was found to be statistically insignificant (*p* = 0.92). However, there was a significant main effect of time (*p* < 0.001), with cfDNA concentrations increasing immediately post-exercise in both cohorts after the first (*p* < 0.001) and the second run (*p* = 0.05). The cfDNA concentrations immediately after the second run were lower compared to those after the first run in both groups (*p* < 0.001).

#### 3.4.6 Assessment of post-exercise pain intensities

**FIGURE 8:**
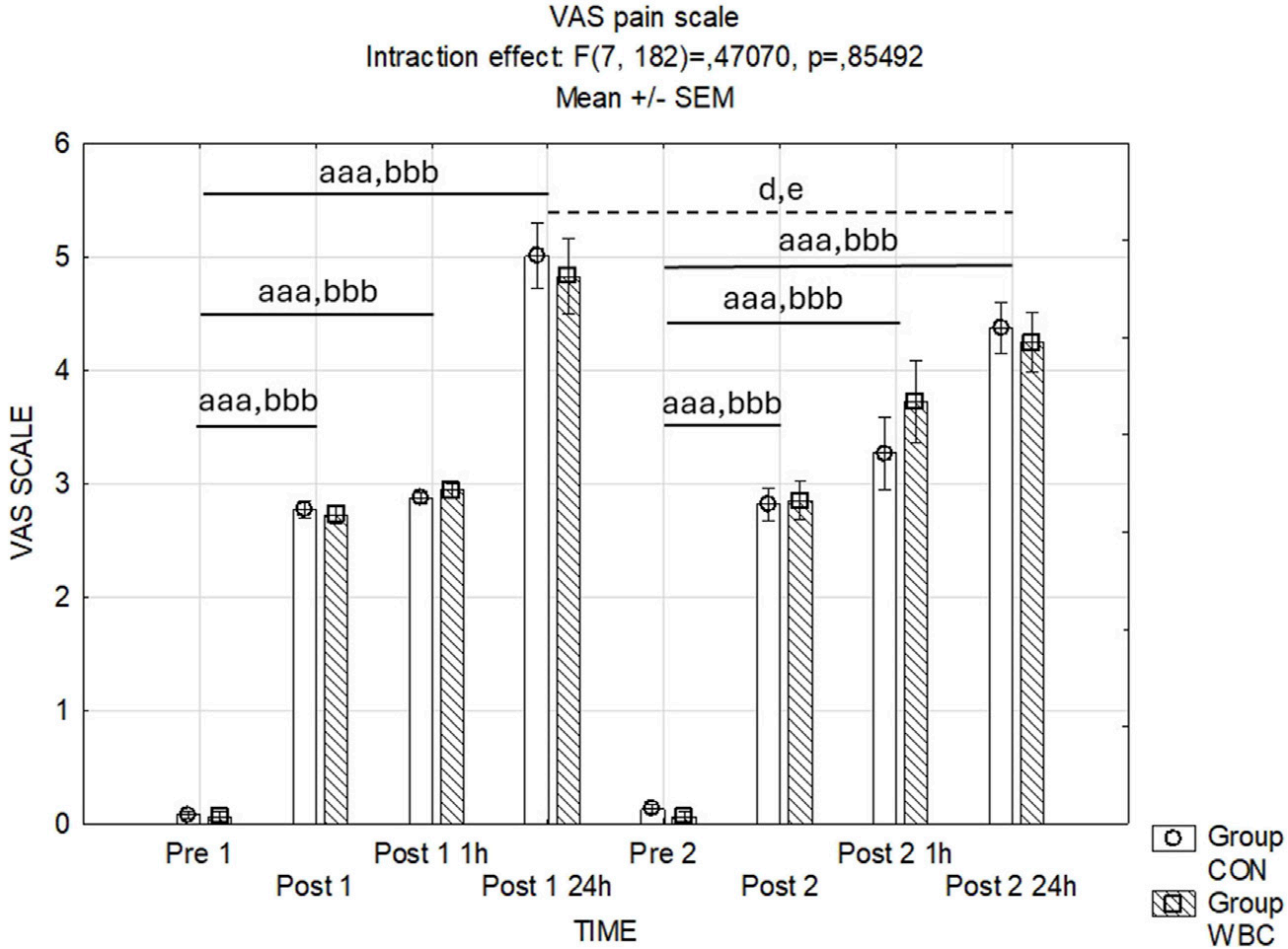
Assessment of post-exercise pain intensities. VAS pain scale values (mean ± SEM) for the control (CON) and whole-body cryotherapy (WBC) groups was measured at the following time points: before the first run (Pre 1), 5 min after the first run (Post 1), 1 h after the first run (Post 1 1 h), 24 h after the first run (Post 1 24 h), before the second run (Pre 2), 5 min after the second run (Post 2), 1 h after the second run (Post 2 1 h), and 24 h after the second run (Post 2 24 h). aaa *p* ≤ 0.001 from corresponding pre-exercise values in CON group; bbb *p* ≤ 0.001 from corresponding pre-exercise values in WBC group; d *p* < 0.05 from corresponding Post 1 24 h in CON Group; e *p* < 0.05 from corresponding Post 1 24 h in WBC group.

Figure 8 illustrates the recorded pain intensities following the squat exercises. The analysis of variance for pain scores post-exercise revealed no statistically significant interaction effect between time points and treatment groups (*p* = 0.85). However, a significant main effect of time was observed (*p* < 0.001), indicating an escalation in reported pain scores subsequent to the exercise, with peak levels occurring at 24 h post-exercise.

## 4 Discussion

The results of this study suggest that the use of whole-body cryotherapy prior to eccentric exercise may confer a protective benefit against post-exercise muscle damage.

In our study, WBC augmented the recognized effect of adaptation to repeated eccentric exercise (Park and Lee, 2015) as demonstrated by a smaller rise in blood myoglobin concentration and creatine kinase activity following the second exercise bout in both groups. This effect was more pronounced in the WBC-treated group. These findings align with the notion that inadequate muscle adaptation to eccentric exercise can lead to more severe myocyte damage (Hyldahl et al., 2017; Chen et al., 2019; Margaritelis et al., 2021). These results suggest a common mechanism underlying the regulation of eccentric work muscle damage by preceding it by exercise and WBC.

Exceeding the tolerance of physical load during muscle work may lead to mechanical damage to cellular structures, including the sarcolemma, which results in an increase in its permeability and the entry of, among others, muscular proteins into the blood. Myoglobin and creatine kinase are specific intracellular proteins that serve as primary biochemical markers indicative of muscle cell damage (Brancaccio et al., 2010; Chalchat et al., 2022). Resistance exercise increases myoglobin concentration and CK activity in both men and women, with women showing a faster inflammatory response and greater prolonged damage response (Heavens et al., 2014). The reduced blood levels of myoglobin and CK activity we observed in the WBC group after a second downhill run, in comparison to the control group, suggest that repeated cryogenic exposure is beneficial for maintaining the structural integrity of myocyte membranes subjected to stretching.

Our records show the greatest changes in blood levels of myoglobin and creatine kinase at time points typical for responses to eccentric exercise (Chalchat et al., 2022). In contrast, our study likely lacks an appropriate time point to capture the post-exercise increase in blood lactate dehydrogenase activity because the activity of this enzyme in serum increases after a longer period than monitored in our study (Chalchat et al., 2022).

The damage to active myocytes may stem from both mechanical and metabolic stress, the latter contributing to oxidative stress due to the excessive production of reactive oxygen species (Hody et al., 2019). Evidence suggests that neutrophil infiltration following eccentric exercise plays a role in muscle damage, inflammatory processes, and delayed-onset muscle soreness—DOMS (Kanda et al., 2013; Hody et al., 2019).

The potential benefits of cryotherapy for enhancing muscle regeneration, as indicated by prior research (Lombardi et al., 2017), might involve the mitigation of reactive oxygen species overproduction resulting from both metabolic stress and neutrophil infiltration post-exercise, thus leading to reduced inflammation and muscle soreness. However, our findings do not entirely support this.

The absence of marked differences in plasma cfDNA concentrations and post-exercise pain intensities between the control and WBC groups implies that WBC’s impact may be more specific to certain indicators of muscle damage. This selective response suggests that WBC may chiefly improve aspects of muscle recovery directly linked to muscular insult rather than inflammation post-exercise in women (Costello et al., 2012; Vieira Ramos et al., 2016).

A possible mediator in the cellular response to low temperatures could be the cold-inducible RNA-binding protein (CIRP), which can post-transcriptionally regulate genes involved in the regulation of metabolism, DNA repair or redox balance (Zhong et al., 2021) as well as stress RNA-binding motif protein 3 (RBM3) involved in the response to low-temperature (Gibson et al., 2017), activated during rapid changes in temperature and in hypoxic conditions. In muscle cells, an elevation in RBM3 concentration may inhibit necrosis and apoptosis as a response to an increase in reactive oxygen species levels (Ferry et al., 2011). It supports the theory that cryotherapy may help reduce cellular breakdown and expedite the removal of debris, thus promoting a more efficient muscle repair (Rose et al., 2017). However, this requires detailed research.

Our finding of rapid release of cfDNA during eccentric exercise in the minute range is highly indicative of a fast, active DNA-release mechanism from immune or endothelial cells, regardless of the use of WBC but as an effect of physical exercise. Amongst the DNA-release mechanisms of cells, NETosis is a vital mechanism that leads to rapid releases of DNA from neutrophils in conjunction with activated thrombocytes within minutes, leaving the releasing cell alive (Neuberger and Simon, 2022). This may explain the equally intense pain in both groups, after both rounds of eccentric exercises, caused by inflammation of similar intensity. NETosis, among other things, involves the generation of reactive oxygen species (Matoszka et al., 2012). In previous studies in women, 10 min after eccentric exercise, an increase in the content of neutrophils and lymphocytes was found, with a simultaneous increase in lipid oxidation (Wiecek et al., 2017). Unfortunately, now we did not examine changes in the leukocyte profile after eccentric exercise. We also did not investigate oxidative damage to macromolecules. However, previously noted were both an increase in neutrophil oxidative burst activity after efforts causing damage to myocytes (Kudoh et al., 2014) as well as no changes in this indicator despite the significant increase in the content of neutrophils correlated with an increase in the concentration of myoglobin and creatine kinase in the blood after 45 min of exercise (downhill running) at an intensity of 60% VO_2_max (Peake et al., 2005).

The amount of oxygen consumption during exercise, is generally lower in women compared to men when normalized to body weight, which, along with sex differences in substrate utilization during exercise, could also affect muscle damage and repair mechanisms (Santisteban et al., 2022). Similarly to other studies (Wiecek et al., 2017), we did not find in women any changes in antioxidant defense after eccentric exercise, measured by uric acid levels, regardless of whether WBC was used.

This is a surprising result. According to previous results, an increase in antioxidant potential after WBC was expected, although previous studies included mainly men who manifested, among other things, after a series of WBC treatments an increase in antioxidant defense by increasing the concentration of GSH (Wojciak et al., 2024) an increase in the activity of antioxidant enzymes (superoxide dismutase) (Wojciak et al., 2020) and nitric oxide synthase (Wiecek et al., 2021).WBC treatments performed in men during muscle-damaging activities reduced the generation of reactive oxygen species (H_2_O_2_ and NO) and pro-inflammatory proteins (Zembron-Lacny et al., 2020).

The probable cause of the different results are gender differences in the pro-oxidant-antioxidant balance. Although it is indicated that, in general, women have a higher antioxidant potential than men (Kander et al., 2017), however, the concentration of uric acid (one of the antioxidants) is higher in men (Wiecek et al., 2017). At the same time, during eccentric exercise, unlike men, women generate more reactive oxygen species (Wiecek et al., 2017), which may reduce the antioxidant effect of WBC use. However, a full interpretation is not possible, due to the limitation of the determination of oxidative stress markers in our studies only to uric acid.

The authors indicate that cryotherapy treatments aimed at accelerating recovery must be applied within a sufficiently short time frame to inhibit the cell-damaging response. In this context, the use of cryotherapy before exercise that damages muscles may prove beneficial. This underlines the complexity of metabolic responses to WBC and suggests that different mechanisms, such as reduced oxidative stress or altered adrenergic activation, might play roles under varying physiological conditions (Christmas et al., 2018)

The lack of difference in pain assessment between groups post-exercise, contrary to expectations, might indicate that the analgesic effects of WBC are more pronounced with either more frequent sessions or different exercise modalities. This observation aligns with the findings from Jastrząbek et al. (2013) and suggests a nuanced interaction between cryotherapy protocols, pain perception, and exercise type.

The current study extends the understanding of gender-specific responses to WBC, addressing a significant gap in the literature, which has predominantly focused on male participants. This research corroborates and expands upon findings from Ferreira-Junior et al. (2014), by illustrating that pre-exercise cryotherapy could be an effective strategy for enhancing recovery and reducing exercise-induced muscle damage in young female.

In considering the influence of various factors on exercise responses and reactions to cryogenic temperatures—including somatic structure, health status, diet, physical performance, and exercise intensity—we endeavored to either eliminate or control these variables. There were no notable differences in initial body composition, hematological measures, or graded exercise test outcomes between the control group and the whole-body cryotherapy group. Subsequent to the initial exercise session, our evaluation of biomarkers related to muscle disruption did not show any significant divergence between the two groups. To confirm that exercise intensity remained within aerobic thresholds, lactate concentrations were measured before exercising and 3 min post-exercise, substantiating the predominance of aerobic metabolism during the activity. Participants adhered to a consistent diet for the duration of the study. As the menstrual cycle did not affect the levels of muscle damage and inflammatory markers in the blood following eccentric exercise, women were included in the study at random phases of their menstrual cycle and participated for four consecutive weeks.

One limitation of our study is the small sample size, which may affect the generalizability of our findings. Additionally, the study did not include an examination of leukocyte profiles or measurements of inflammatory and oxidative stress markers, which would have provided a more comprehensive understanding of the physiological responses to exercise and cryotherapy.

In conclusion, while this study reinforces the beneficial role of WBC in reducing specific muscle damage markers following eccentric exercise, it also highlights the necessity for further research. To understand the mechanism accompanying the reduction in CK and myoglobin levels after a downhill run preceded by 20 WBC treatments, it would be necessary to examine the role of inflammatory mediators involved both in muscle regeneration and the cellular response to cryotherapy (Dugué, 2015; Zuo et al., 2019). Future studies should explore the optimal timing and frequency of WBC sessions, its long-term effects on different types of muscle fibers, and its efficacy across diverse athletic and clinical populations, including both genders to fully harness its therapeutic potential.

## Data Availability

The raw data supporting the conclusion of this article will be made available by the authors, without undue reservation.
